# A Common Iba1 Antibody Labels Vasopressin Neurons in Mice

**DOI:** 10.1523/ENEURO.0323-25.2025

**Published:** 2026-01-30

**Authors:** Hannah D. Lichtenstein, Faith Kamau, Shaina McGrath, Javier E. Stern, Jessica L. Bolton

**Affiliations:** Neuroscience Institute and the Center for Neuroinflammation and Cardiometabolic Diseases, Georgia State University, Atlanta, Georgia 30303

**Keywords:** CX3CR1, Iba1, immunohistochemistry, microglia, P2RY12, vasopressin

## Abstract

There are a wide variety of commercially available antibodies for labeling microglial cells based on different protein targets, as well as antibodies for the same protein target made in different species. While this array of targets and hosts allows for flexibility in immunohistochemical experiments, it is important to validate that different antibodies provide comparable and accurate immunodetection prior to experimental data collection. We found that a commercially available anti-Iba1 antibody, made in goat, produces irregular staining patterns in specific regions of the mouse brain in both sexes, prompting a further investigation into the phenomenon. This Iba1-goat antibody displayed increased numbers of labeled cells when compared with expression of a CX3CR1-GFP reporter and IHC detection of P2RY12, two common microglial markers. Furthermore, immunodetection by other common anti-Iba1 antibodies made in rabbit and chicken did not display the excessive cell labeling when compared with the CX3CR1-GFP reporter. Upon further investigation, this Iba1-goat antibody was observed to highly colocalize with vasopressin neurons in the paraventricular nucleus of the hypothalamus (PVN) and the supraoptic nucleus of the hypothalamus (SON), the two main sites of vasopressin production in the brain. Other anti-Iba1 antibodies made in other species did not show this same colocalization with vasopressin. Finally, this effect was species-specific, as Wistar rats did not display erroneous cell labeling by the Iba1-goat antibody. In sum, the present study employs both qualitative and quantitative data to highlight the importance of validating antibody efficacy and specificity in a region- and species-specific manner.

## Significance Statement

Microglia are the primary immune cells of the brain and are involved in many neurodevelopmental, as well as neurodegenerative, processes, thus making the study of microglia an important area of neuroscience research. There are a wide array of antibodies available to label microglia. Specific detection of microglia using immunohistochemistry is crucial for understanding differences in cell density, morphology, and interactions with other cells in various contexts. In the present study, a common anti-Iba1 antibody made in goat was found to display erroneous labeling of vasopressin neurons in specific regions of the mouse brain, inconsistent with other microglial markers, which emphasizes the importance of validating antibody efficacy and specificity in a region- and species-specific manner prior to beginning experimental data collection.

## Introduction

Microglia are the resident immune cells of the brain and are involved in many important neurodevelopmental and neurodegenerative processes (Paolicelli et al., 2011; Schafer et al., 2012; Lenz et al., 2013; Miyamoto et al., 2016; Nelson et al., 2021; Bolton et al., 2022). Thus, having the tools to visualize these cells in the brain is important for advancing neuroscience research, whether via transgenic expression of fluorescent reporters (for review, see Zhao et al., 2019) or immunohistochemistry. Some of the most commonly used immunohistochemical targets for visualizing microglia are ionized calcium-binding adapter molecule 1 (Iba1), purinergic receptor P2Y12 (P2RY12), transmembrane protein 119 (TMEM119), and the fractalkine receptor (CX3CR1), all of which have varying levels of specificity for microglia versus macrophages (for review, see Jurga et al., 2020).

Importantly, the signal intensity of these microglial markers can fluctuate depending on the age of an organism, due to the changes in gene expression within microglia throughout the lifespan (Hammond et al., 2019; Gómez Morillas et al., 2021). Iba1, or ionized calcium-binding adaptor molecule 1, has been shown to be expressed specifically in monocyte/macrophage cell lineages and functions as an adapter molecule that mediates calcium signals in these cells (Ito et al., 1998; Okere and Kaba, 2000). While other microglial markers, like CR3 or ED2, are only expressed in subsets of microglia, Iba1 is expressed by all microglial subpopulations, making it the most popular pan-microglial marker (Ito et al., 1998; Shapiro et al., 2009). Specificity is another factor that comes into play when choosing a microglial marker: while Iba1 may be the most popular target for visualizing microglia via IHC, this marker is also expressed by macrophages, whether brain-resident or infiltrating from the periphery (Ohsawa et al., 2004), which can sometimes localize quite close to microglia (Cronk et al., 2018).

Due to the nature of these tools and techniques, it is good scientific practice to use multiple markers for microglia when embarking on a research project that uses specific antibodies for the first time to validate the efficacy of any one antibody. Not only do different microglial markers have their unique limitations, but issues with reproducibility in general can also arise due to lot-to-lot variability, even when prepared from the same donor animal (Meliopoulos and Schultz-Cherry, 2018). Donor animals of different species, such as goat, rabbit, and chicken, allow for the use of multiple targets within one experiment, as antibodies developed within one species will typically not interfere with the binding of antibodies from other species. Thus, commonly used antibodies, such as Iba1, are typically manufactured in a variety of species, so that researchers are able to conduct experiments with different combinations of molecular targets in one tissue sample.

We recently observed that a common anti-Iba1 antibody, made in goat, produced irregular staining patterns in select regions in the mouse brain, such as the paraventricular nucleus of the hypothalamus (PVN) and the supraoptic nucleus (SON): Microglia were labeled by Iba1, but so were cells that had a nonmicroglial morphology and colocalized with vasopressin. When alternative microglial markers were used, such as P2RY12 and CX3CR1-GFP, they did not colocalize with vasopressin. Furthermore, when different Iba1 antibody species were used, such as those made in chicken and rabbit, the unusual staining was not detected. The present study outlines the observed effects both qualitatively and quantitatively, highlighting the importance of validating antibody efficacy and specificity in a region- and species-specific manner prior to beginning experiments.

## Materials and Methods

### Animals

The mice used in this study were immature [postnatal day (P)8] or adult (P60–90) males and females from either a CX3CR1-GFP+/− reporter line (strain #005582, The Jackson Laboratory, RRID:IMSR_JAX:005582; where transgenic microglial reporters were included) or “wild-type” mice (i.e., Cre−/−) from a CX3CR1-BAC-Cre mouse line (MGI: 5311737; RRID: MMRRC: 036395-UCD), both from a C57BL/6 background (although the CX3CR1-GFP mice are the C57BL/6J substrain, whereas the CX3CR1-BAC-Cre mice are the C57BL/6N substrain). The rats used in this study were adult male Wistar rats (RccHan:WIST, Envigo, RRID:RGD_13508588). Mice and rats were housed under a 12 h light/dark cycle with free access to food and water. All experiments were performed in accordance with National Institutes of Health (NIH) guidelines and were approved by the Georgia State University Animal Care and Use Committee.

### Experimental design

The accuracy of the goat anti-Iba1 antibody was analyzed in CX3CR1-GFP+/− and wild-type mice. Animals were killed with Euthasol (Patterson Veterinary, VIRBAC Animal Health) and transcardially perfused with ice-cold 1× phosphate-buffered saline (PBS) followed by 4% paraformaldehyde. Perfused brains of CX3CR1-GFP+/− mice were then post-fixed in 4% paraformaldehyde in 0.1 M PBS for 4–6 h before cryopreserving in 15% sucrose solution overnight followed by 25% (for pups) or 30% (for adults) sucrose solution until brains sank. Brains were frozen by dipping into 2-methylbutane for ∼30 s and were stored in a −80°C freezer. Pups were weaned on P21 into standard, same-sex cages of 2–5 mice and killed with the same procedure as described before on P60.

### Immunohistochemistry (IHC)

Brains of CX3CR1-GFP+/− and wild-type mice were coronally sectioned into 25-µm-thick slices (1:4 series of the PVN and SON) using a Leica CM1860 cryostat (RRID: SCR_025772) and stored in an anti-freeze solution in a −20°C freezer. For IHC, floating brain sections were washed several times (3 × 5 min) with PBS-T (PBS containing 0.3% Triton X-100; Thermo Fisher Scientific catalog #AC215682500; pH = 7.4) at room temperature (RT). Tissues were then allowed to permeabilize in 0.09% H_2_O_2_ in PBS-T for 20 min at RT. Sections were washed several times with PBS-T and then incubated in blocking solution containing 5% normal donkey serum (NDS; Jackson ImmunoResearch catalog #017-000-121, RRID: 2337258) for 1 h at RT to prevent nonspecific binding. Sections were then incubated in primary antibody solution overnight at 4°C. The next morning, sections were washed in PBS-T several times and incubated in secondary antibody solution (Table 1) for 3 h at RT. After repeated PBS-T washes, free-floating sections were counterstained with DAPI for 1 min before mounting on gelatin-coated slides and coverslipping with Fluoromount-G Mounting Medium (Thermo Fisher Scientific catalog #00-4958-02, RRID: SCR_015961). Immunostaining P8 brain sections with anti-Iba1-chicken and anti-Iba1-rabbit required antigen retrieval with Tris-EDTA buffer at 90°C for 3 min prior to the blocking step before continuing with the protocol described above.

**Table 1.** Summary of antibodies used for immunohistochemical experiments

Primary antibody	Concentration and buffer	Secondary antibody	Concentration and buffer
Goat anti-Iba1 (Iba1-goat), Fujifilm WAKO Pure Chemical Corporation catalog #011-27991, RRID: AB_2935833 Lots: SKK1868, LEG4278, WTQ1615	1:1,000 2% NDS, 0.3% Triton X-100	Donkey anti-goat IgG-568, Thermo Fisher Scientific catalog #A11057, RRID: AB_2534104, Lot 23044269 Donkey anti-goat IgG-647, Jackson ImmunoResearch catalog #705-607-003, RRID: AB_2340439	1:500 2% NDS, 0.3% Triton X-100
Chicken anti-Iba1 (Iba1-chicken), Synaptic Systems catalog #234-009, RRID: AB_2891282 Lots: 1-11, 1-16	1:2,000 2% NDS, 0.3% Triton X-100	Alexa Fluor-568 AffiniPure Donkey anti-chicken IgG, Thermo Fisher Scientific catalog #A78950, RRID: AB_291072, Lot 2622381 Alexa Fluor-647 Donkey anti-chicken IgG, Jackson ImmunoResearch catalog #703-605-155, RRID: AB_2340379, Lots 160355, 2540901	1:1,000 2% NDS, 0.3% Triton X-100
Rabbit anti-Iba1 (Iba1-rabbit), Fujifilm WAKO Pure Chemical Corporation catalog #019-19741, RRID: AB_839504 Lot: SEP6150	1:2,000 2% NDS, 0.3% Triton X-100	Donkey anti-rabbit IgG, Alexa Fluor-568, Thermo Fisher Scientific catalog #A10042, RRID: AB_2534017, Lots 2306809, 2540901	1:1,000 2% NDS, 0.3% Triton X-100
Rabbit anti-P2RY12, AnaSpec; EGT Group catalog #AS-55043A, RRID: AB_2298886 Lots: WF0501, WTQ1615	1:2,000 2% NDS, 0.3% Triton X-100	Alexa Fluor-647 AffiniPure donkey anti-rabbit IgG, Jackson ImmunoResearch catalog #711-605-152, RRID: AB_2492288, Lots 167518, 173915	1:1,000 2% NDS, 0.3% Triton X-100
Rabbit anti-vasopressin–neurophysin (VP–NP), Dr. Alan Robinson, University of Pittsburgh catalog #rn2, RRID: AB_2751000	1:20,000 2% NDS, 0.3% Triton X-100	Donkey anti-rabbit IgG, Alexa Fluor-568, Thermo Fisher Scientific catalog #A10042, RRID: AB_2534017, Lots 2306809, 2540901 Donkey anti-rabbit IgG, Alexa Fluor-488, Thermo Fisher Scientific catalog #A-21206, RRID: AB_2535792, Lots 2289872, 2873188	1:500 2% NDS, 0.3% Triton X-100
Mouse anti-oxytocin–neurophysin (OT–NP), Ben-Barak et al. (1985), catalog #PS36, RRID: AB_2315027	1:20,000 2% NDS, 0.3% Triton X-100	Donkey anti-mouse IgG, Alexa Fluor-568, Thermo Fisher Scientific catalog #A10037, RRID: AB_1180865, Lot 2555709	1:500 2% NDS, 0.3% Triton X-100

From left to right: primary antibody, associated concentration and buffer; secondary antibody, associated concentration and buffer.

Immunostaining for vasopressin–neurophysin (VP–NP; Verbalis and Robinson, 1983) and oxytocin–neurophysin (OT–NP; Ben-Barak et al., 1985) expression differed slightly from the protocol described above. After incubation in blocking solution of 5% NDS for 1 h at RT to prevent nonspecific binding, sections were incubated with the primary antibody solution for anti-VP–NP-rabbit and/or anti-OT–NP-mouse overnight at 4°C. The next day, sections were washed in PBS-T several times and incubated in secondary antibody solution (Table 1) for 3 h at RT. After repeated PBS-T washes, tissues were incubated in blocking solution containing 5% NDS for 1 h at RT. Sections were then incubated with the primary antibody solution for anti-Iba1-goat overnight. The next day, sections were washed several times with PBS-T and then incubated with the secondary antibody solution (Table 1) for 3 h at RT. The protocol then continues as described above. Methodological details, catalog #, and RRID for each antibody are specified in Table 1.

### Analysis

Images for the comparison of goat, chicken, and rabbit anti-Iba1 antibodies were taken with an Olympus BX41 Epifluorescent Clinical Microscope (RRID: SCR_027355). All other images were collected using a Zeiss LSM 780 Confocal Laser Scanning Microscope (RRID: SCR_020922) with a 20× objective. A total of 22 *Z*-stack images were acquired at 0.5 µm intervals. The image frame was digitized at 16-bit using a 1,024 × 1,024-pixel frame size. Images for the Wistar rats used in Figure 5 were collected with the same settings, and the inset was captured with a 63× objective. Using Fiji (RRID: SCR_002285), an ROI was manually drawn around the PVN or SON, and then the number and colocalization of Iba1+ microglia, P2RY12+ microglia, VP+, OT+, and GFP reporter+ microglia were counted manually. Statistical differences were assessed using GraphPad Prism software (RRID: SCR_002798). Each group consisted of *n* = 3 animals (comprised of both sexes). Two-way ANOVA tests were used to analyze the accuracy of the goat anti-Iba1 antibody using colocalization with the different microglial markers as the independent variables. Significant interactions were followed by Šídák post hoc tests. The significance level was set to 0.05 for all tests, and data are represented as mean ± SEM. All experiments were performed blindly without prior knowledge of the experimental group.

## Results

### Iba1-goat antibody displays increased cell labeling compared with P2RY12 and CX3CR1-GFP expression, in both pups and adult mice, in the PVN and SON

**Figure 1.**
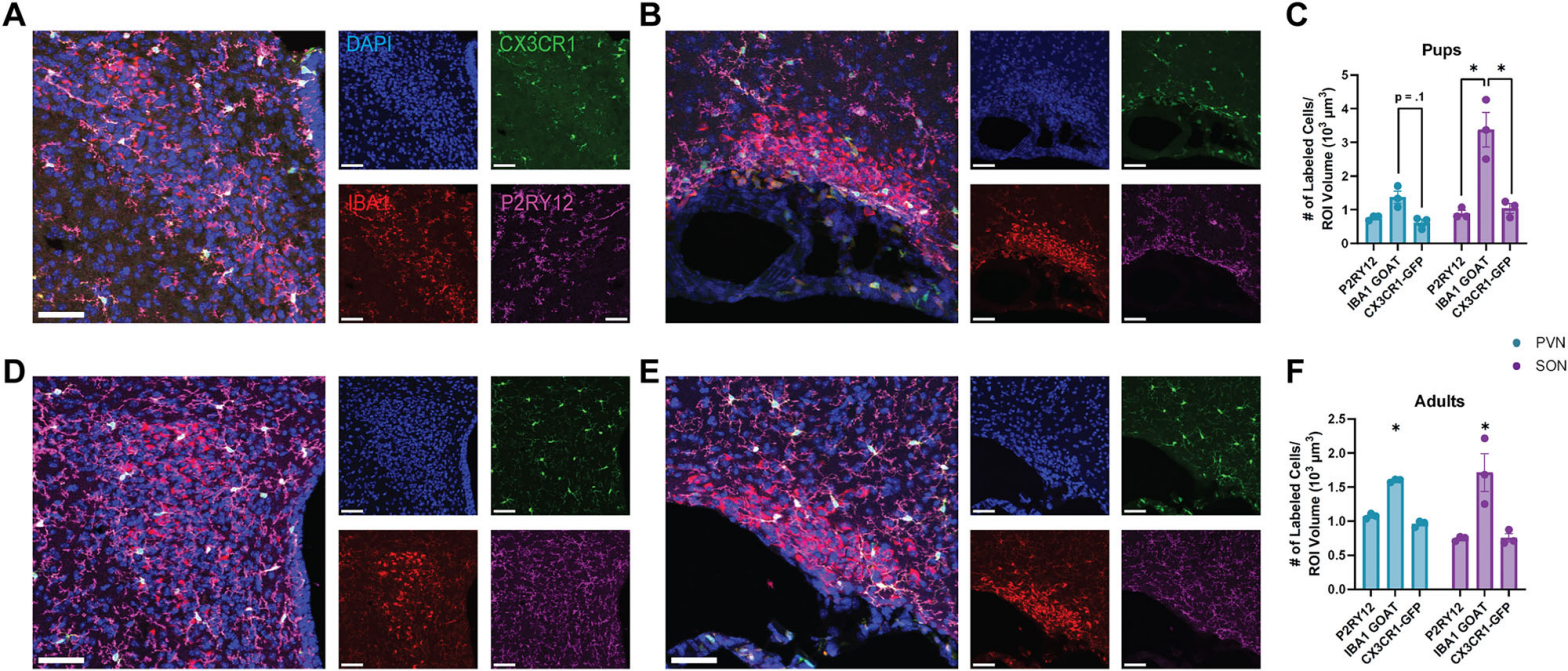
Iba1-goat displays increased cell labeling compared with P2RY12 and CX3CR1-GFP expression, in both pups and adult mice, in the PVN and SON. ***A, B***, Representative images of the PVN (***A***) and SON (***B***) of pups. ***C***, In the PVN, Iba1-goat antibody displayed a slight increase in the number of cells labeled compared with CX3CR1-GFP, and in the SON, Iba1-goat displayed a significant increase in the number of cells labeled within the region of interest compared with both P2RY12 and CX3CR1-GFP (significant brain region–microglial marker interaction; *F*_(2,4)_ = 14.32, *p* = 0.02; post hoc tests, *p*_PVN Iba1-goat vs CX3CR1_ = 0.1, *p*_PVN Iba1-goat vs P2RY12_ = 0.2, *p*_SON Iba1-goat vs P2RY12_ = 0.002, *p*_SON Iba1-goat vs CX3CR1-GFP_ = 0.002). ***D, E***, Representative images of the PVN (***D***) and SON (***E***) of adults. ***F***, In the PVN and SON, Iba1-goat antibody displayed an increase in the number of cells labeled compared with both P2RY12 and CX3CR1-GFP (significant main effect of Microglial Marker; *F*_(2,8)_ = 23.45, *p* = 0.0005). This effect was not seen in other brain regions (Fig. 1-1). Data are mean ± SEM; *p < 0.05. Scale bar, 20 µm. Blue, DAPI; green, CX3CR1-GFP; red, Iba1-goat; magenta, P2RY12.

Using CX3CR1-GFP+/− mice that have transgenic fluorescent reporters in microglia, we examined the difference in density of cell labeling compared with Iba1-goat and P2RY12, two microglial markers commonly used in IHC, in both pups (Fig. 1*A*,*B*) and adult mice (Fig. 1*D*,*E*). Across pups and adults in both the PVN and SON, expression of CX3CR1-GFP and P2RY12 showed similar densities of labeled cells, whereas Iba1-goat resulted in a significantly increased density of labeled cells. In pups, there was a trending increase in the density of Iba1-goat+ cells compared with CX3CR1-GFP+ cells in the PVN (Fig. 1*A*,*C*), and a significant increase in the density of Iba1-goat-labeled cells compared with both CX3CR1-GFP+ and P2RY12+ cells in the SON (Fig. 1*B*,*C*). These results suggest that the effect may be stronger in the SON than in the PVN at this age. In adults, both the PVN and SON had a significant increase in the density of Iba1-goat+ cells compared with both CX3CR1-GFP+ and P2RY12+ cells (Fig. 1*D*,*F*), suggesting a more prominent effect in adulthood. Interestingly, this effect was only evident in the PVN and SON, as other brain regions like the hippocampus and cortex did not display excessive cell labeling (Fig. 1-1).

10.1523/ENEURO.0323-25.2025.f1-1 Figure 1-1 **Excessive Iba1-goat staining is confined to specific regions of the mouse brain.** A-B) Representative images of the parietal cortex in A) pups and B) adults. C-D) Representative images of the CA1 of the hippocampus in (C) pups and (D) adults. Scale bars = 20 μm, blue = DAPI, green = CX3CR1, red = Iba1-goat, magenta = P2RY12. Download Figure 1-1, TIF file.

### Iba1-goat displays excessive labeling of nonmicroglial cells compared with Iba1-rabbit and Iba1-chicken, in both pups and adult mice, in the PVN and SON

**Figure 2.**
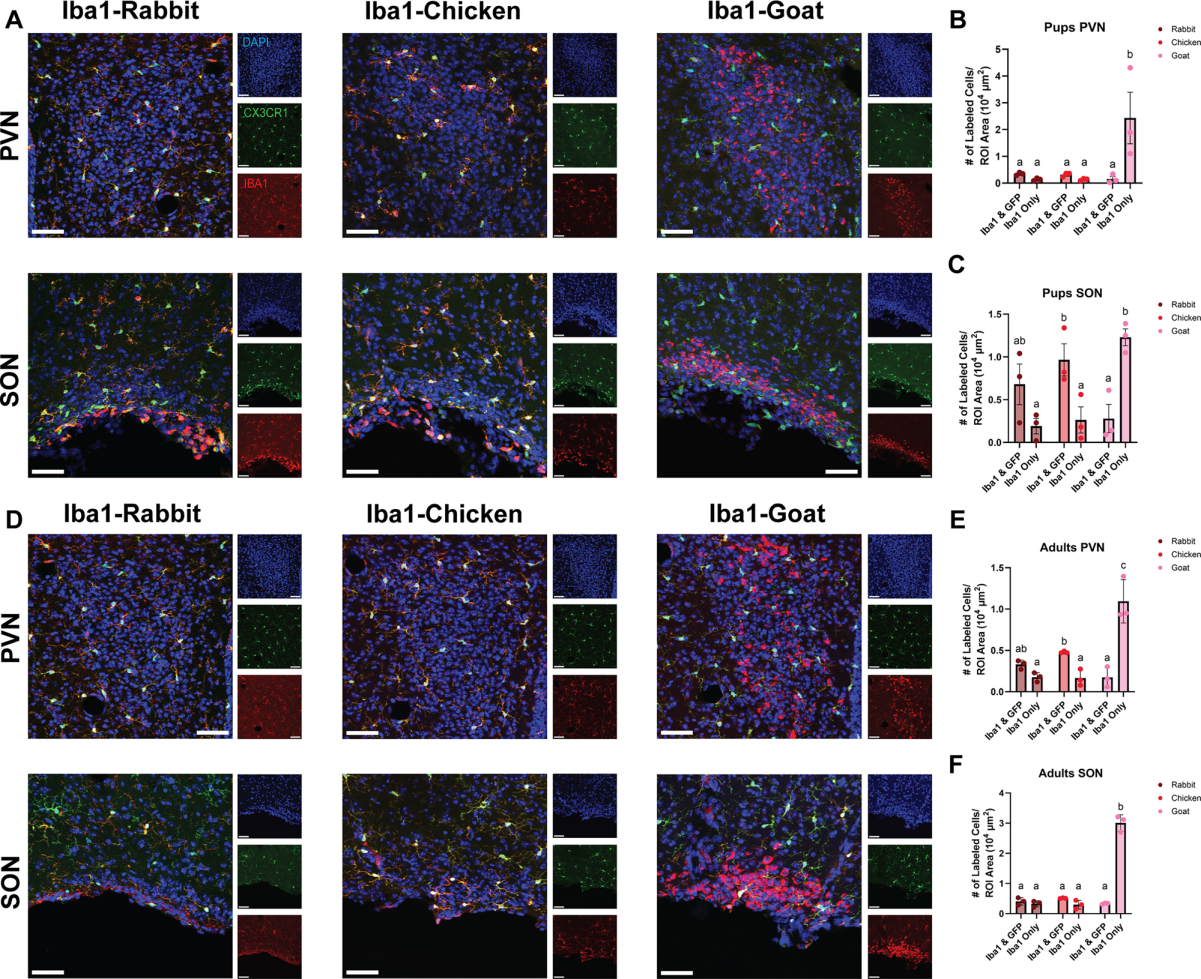
Iba1-goat displays excessive labeling of nonmicroglial cells compared with Iba1-rabbit and Iba1-chicken, in both pups and adult mice, in the PVN and SON. ***A, D***, Representative images, from left to right, top to bottom, of Iba1-rabbit, Iba1-chicken, and Iba1-goat antibodies immunostaining in the PVN and SON in pups (***A***) and adults (***D***). ***B***, In the PVN of pups, Iba1-goat antibody displayed an increase in the number of cells labeled compared with Iba1-goat antibody colocalized with CX3CR1-GFP within the same tissue, as well as when compared with the number of cells labeled with Iba1-chicken and Iba1-rabbit (significant cell density–Iba1 species interaction; *F*_(2,6)_ = 7.996, *p* = 0.02; post hoc test, *p*_Iba1 only, rabbit vs goat_ = 0.005^b^, *p*_Iba1 only, chicken vs goat_ = 0.005^b^, *p*_Iba1-goat, Iba1 only vs Iba1 and GFP_ = 0.004^b^). ***C***, In the SON of pups, Iba1-goat antibody displayed an increase in the number of cells labeled compared with Iba1-goat and CX3CR1-GFP colocalized within the same tissue, as well as when compared with the number of cells labeled with Iba1-chicken and Iba1-rabbit (significant cell density–Iba1 species interaction; *F*_(2,6)_ = 17.40, *p* = 0.003; post hoc test, *p*_Iba1 only, rabbit vs goat_ = 0.002^b^, *p*_Iba1 only, chicken vs goat_ = 0.003^b^, *p*_Iba1-goat, Iba1 only vs Iba1 and GFP_ = 0.005^b^, *p*_Iba1 and GFP, chicken vs goat_ = 0.03^a^, *p*_chicken, Iba1 only vs Iba1 and GFP_ = 0.02^a^). ***E***, In the PVN of adults, Iba1-goat antibody displayed an increase in the number of cells labeled compared with Iba1-goat and CX3CR1-GFP colocalized within the same tissue, as well as when compared with the number of cells labeled with Iba1-chicken and Iba1-rabbit (significant cell density–Iba1 species interaction; *F*_(2,6)_ = 93.24, *p* < 0.0001; post hoc test, *p*_Iba1 only, rabbit vs goat_ < 0.0001^c^, *p*_Iba1 only, chicken v goat_ < 0.0001^c^, *p*_Iba1-goat, Iba1 only vs Iba1 and GFP_ < 0.0001^c^, *p*_chicken, Iba1 only vs Iba1 and GFP_ = 0.004^b^, *p*_Iba1 and GFP, chicken vs goat_ = 0.04^b^). ***F***, In the SON of adults, Iba1-goat antibody displayed an increase in the number of cells labeled within the region of interest compared with Iba1-goat and CX3CR1-GFP colocalized within the same tissue, as well as when compared with the number of cells labeled with Iba1-chicken and Iba1-rabbit (significant cell density–Iba1 species interaction; *F*_(2,6)_ = 176.44, *p* < 0.0001; post hoc test, *p*_Iba1 only, rabbit vs goat_ < 0.0001^b^, *p*_Iba1 only, chicken vs goat_ < 0.0001^b^, *p*_Iba1-goat, Iba1 only vs Iba1 and GFP_ < 0.0001^b^). Data are mean ± SEM. Scale bar, 20 µm. Blue, DAPI; green, CX3CR1; red, Iba1 from either rabbit (left), chicken (center), or goat (right).

Using the same CX3CR1-GFP+/− mice, we next examined how three different versions of the Iba1 antibody, made in various host species, labeled microglia when compared with CX3CR1-GFP (Fig. 2*A*,*D*). There was a significant increase in the density of Iba1-goat+ cells in the PVN of pups when compared with other Iba1 species (Fig. 2*B*) and compared with colocalization with CX3CR1-GFP (Fig. 2*B*). In the SON of pups, there was a significant increase in the density of Iba1-goat+ cells when compared with Iba1-rabbit (Fig. 2*C*) or Iba1-chicken (Fig. 2*C*). The density of Iba1-goat+ cells was significantly increased when compared with colocalization with CX3CR1-GFP (Fig. 2*C*), whereas Iba1-chicken+ cells were significantly decreased when compared with colocalization with CX3CR1-GFP (Fig. 2*C*). In the PVN of adults, there was a significant increase in the density of Iba1-goat+ cells when compared with other species of anti-Iba1 (Fig. 2*E*) and compared with colocalization with CX3CR1-GFP (Fig. 2*E*). Iba1-chicken displayed increased colocalization with CX3CR1-GFP+ cells compared with only Iba1+ cells (Fig. 2*E*) and compared with Iba1-goat colocalization with CX3CR1-GFP (Fig. 2*E*). In the SON of adults, there was a significant increase in the density of Iba1-goat+ cells when compared with other Iba1 species (Fig. 2*F*) and compared with colocalization with CX3CR1-GFP (Fig. 2*F*). These findings show that Iba1-goat labels microglial cells and many other cells that are not CX3CR1-GFP+, in both the PVN and SON of pups and adults, suggesting that the Iba1-goat antibody has off-target labeling in certain regions of the mouse brain.

### Iba1-goat displays high colocalization with vasopressin, and to some degree oxytocin, in the PVN and SON of adult mice

**Figure 3.**
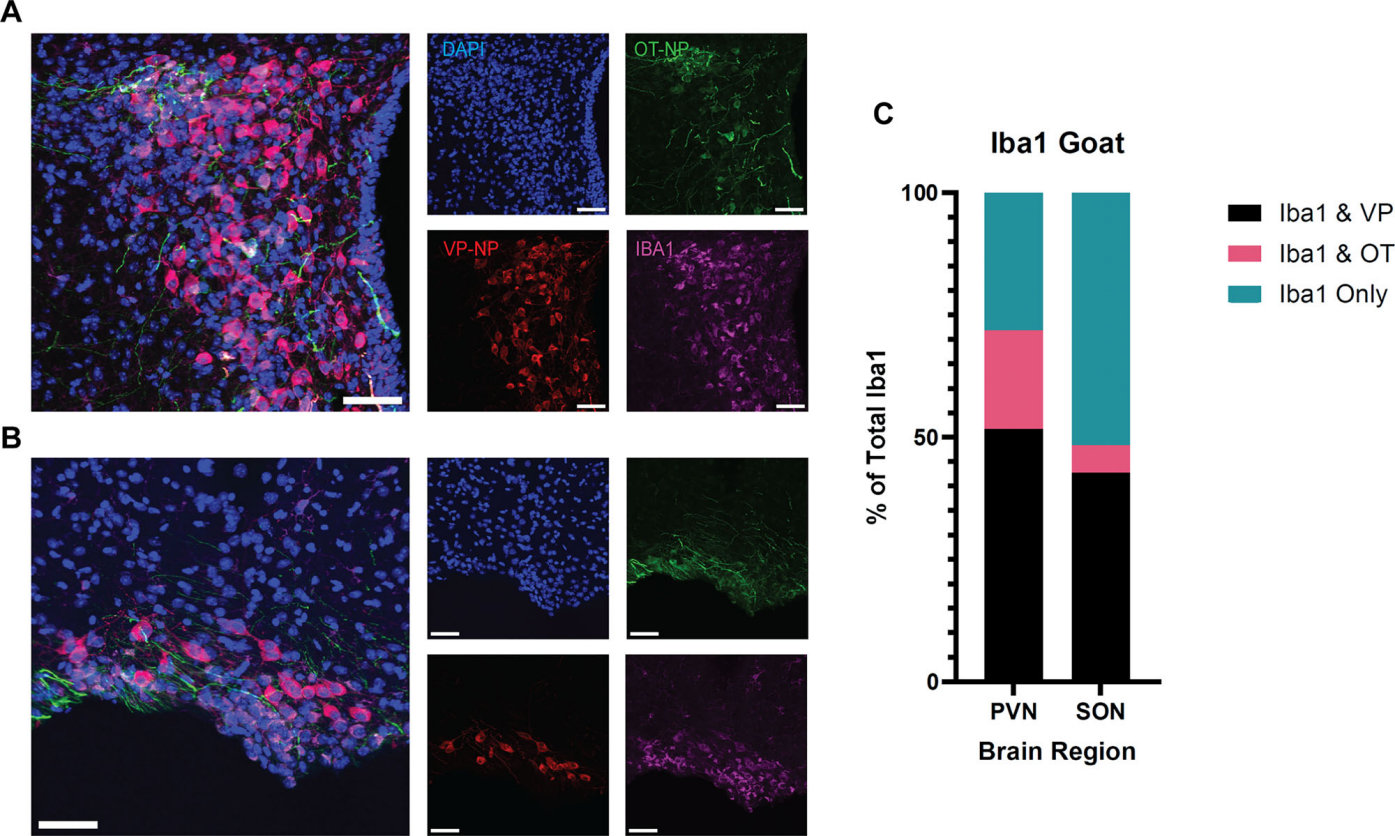
Iba1-goat displays high colocalization with vasopressin, and to some degree oxytocin, in the PVN and SON of adult mice. ***A, B***, Representative images of the PVN (***A***) and SON (***B***). ***C***, Comparison of the percentage of cells labeled by Iba1-goat antibody alone, Iba1-goat colocalized with VP, and Iba1-goat colocalized with OT within the PVN and the SON. 51.7% ± 13.3 of cells in the PVN and 42.7% ± 2.9 of cells in the SON were co-labeled with Iba1-goat and VP. In the PVN, 20.2% ± 12.6 of the cells were co-labeled with Iba1-goat and OT, whereas in the SON, 5.6% ± 0.7 of the cells were co-labeled with Iba1-goat and OT. In the PVN, the remaining cells were labeled with Iba1-goat antibody only, making up 28.1% ± 0.8 of the labeled cells, as opposed to the SON, wherein 51.7% ± 2.2 of the cells were labeled with Iba1-goat only. Colocalization in *z*-stacks can be visualized in Figure 3-1. Data are mean ± SEM. Scale bar, 20 µm. Blue, DAPI; green, VP; red, OT; magenta, Iba1-goat.

In order to better understand the identity of the nonmicroglial cells that are labeled by Iba1-goat, we examined the morphology of the cells and postulated that the Iba1-goat+ nonmicroglial cells appeared to look quite similar to those of principal neurons. Since the localization of this excessive Iba1-goat signal is so strong in the PVN and SON, and these are the main brain regions that produce vasopressin (VP) and oxytocin (OT), we investigated whether or not Iba1-goat colocalized with either of these signals in wild-type C57BL/6 mice (Fig. 3*A*,*B*). When comparing the percentage of Iba1+ cells labeled by Iba1-goat alone, Iba1-goat colocalized with VP, and Iba1-goat colocalized with OT (Fig. 3*C*, Fig. 3-1), we found that ∼40 and 55% of the Iba1-labeled cells in the PVN and the SON, respectively, are co-labeled with VP. In the PVN, ∼20% of the Iba1+ cells are co-labeled with OT, whereas in the SON, <10% of the Iba1+ cells are co-labeled with OT. In the PVN, the remaining Iba1+ cells were labeled with Iba1-goat only, making up ∼25% of the Iba1-labeled cells, compared with the SON, wherein ∼50% of the Iba1+ cells were labeled with Iba1-goat only, likely indicating the number of true microglia/macrophages. These results suggest that although the Iba1-goat antibody does label microglia, it also shows off-target effects, labeling many vasopressin- and oxytocin-expressing neurons in the PVN and SON.

10.1523/ENEURO.0323-25.2025.f3-1 Figure 3-1 **Representative z-stack image sequence to confirm true colocalization between Iba1 and OT and VP.** Corresponds to the PVN samples from Figure 3. Scale bar = 20 μm, blue = DAPI, green = VP, red = OT, magenta = Iba1-goat. White arrowheads represent examples of colocalization between Iba1-goat, and VP and/or OT. Download Figure 3-1, TIF file.

### When compared with Iba1-goat, Iba1-chicken displays little to no colocalization with vasopressin, and high levels of colocalization with CX3CR1-GFP

**Figure 4.**
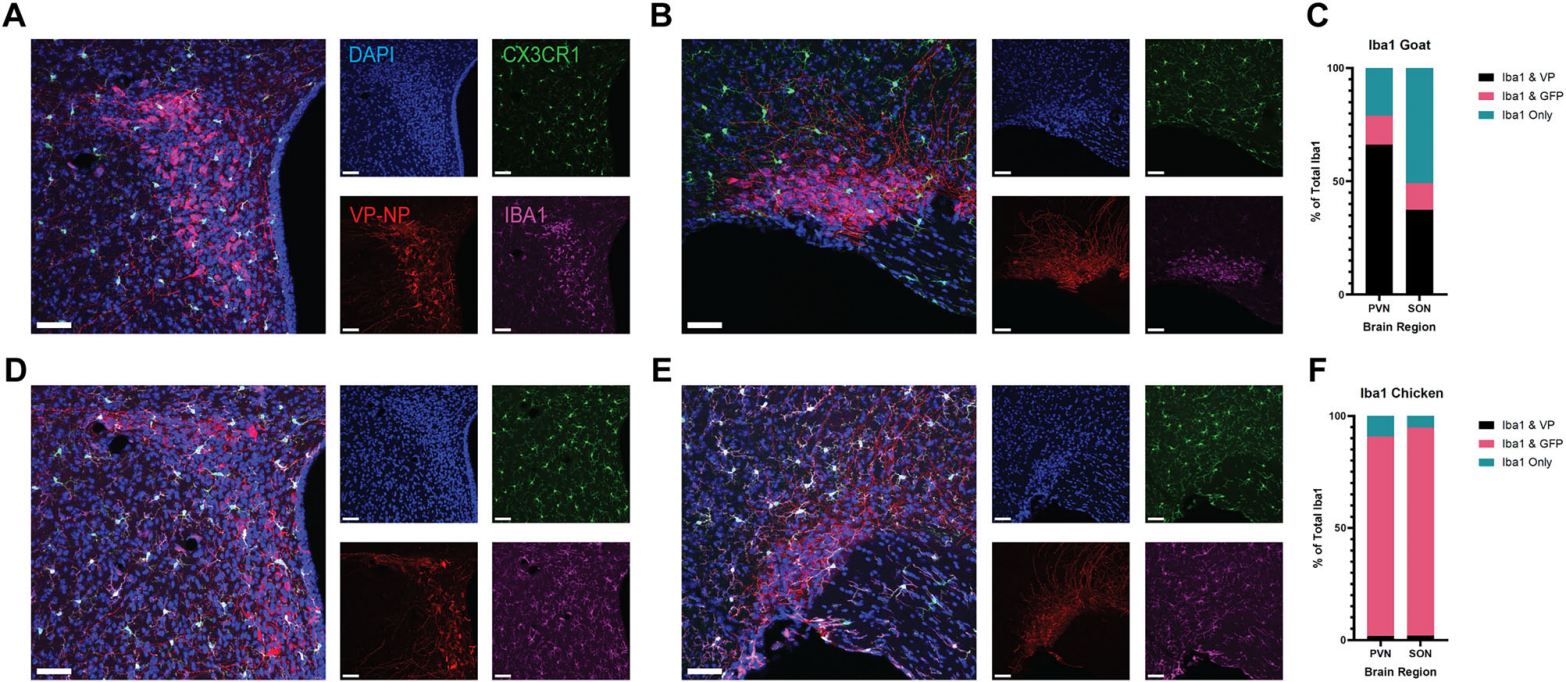
When compared with Iba1-goat, Iba1-chicken displays little to no colocalization with vasopressin and high levels of colocalization with CX3CR1-GFP. ***A, B***, Representative images of the PVN (***A***) and SON (***B***). ***C***, Comparison of the percentage of cells labeled by Iba1-goat antibody alone, Iba1-goat colocalized with CX3CR1-GFP, and Iba1-goat colocalized with VP within the PVN and the SON. 66.2% ± 4.1 of Iba1+ cells in the PVN and 37.4% ± 3.1 of Iba1+ cells in the SON were co-labeled with Iba1-goat and VP. 12.7% ± 1.9 and 11.7% ± 2.5 of Iba1-goat cells were co-labeled with CX3CR1-GFP in both the PVN and the SON, respectively, and the remaining 21.1% ± 4.5 of cells in the PVN and 50.9% ± 2.2 of the cells in the SON were labeled with only Iba1-goat. ***F***, Comparison of the percentage of cells labeled by Iba1-chicken alone, Iba1-chicken colocalized with CX3CR1-GFP, and Iba1-chicken colocalized with VP within the PVN and the SON. 1.8% ± 0.9 and 2.0% ± 1.8 of the Iba1+ cells were labeled by Iba1-chicken colocalized with VP in either the PVN or SON, respectively. 88.8% ± 6.1 of the Ibal+ cells in the PVN and 92.7% ± 5.2 of the cells in the SON were co-labeled with Iba1-chicken and CX3CR1-GFP. The remainder of Iba1+ cells in the PVN (9.7% ± 5.0) and the SON (5.3% ± 5.0) were labeled with only Iba1-chicken. Importantly, this effect is not due to differences in staining robustness of VP+ cells (Fig. 4-1). Data are mean ± SEM. Scale bar, 20 µm. Blue, DAPI; green, CX3CR1-GFP; red, VP; magenta, Iba1-goat (***A, B***) or Iba1-chicken (***D, E***).

After observing considerable colocalization of Iba1-goat with VP, we wanted to confirm whether or not this pattern was observed with Iba1-chicken (Fig. 4*A*,*B*). Using CX3CR1-GFP+/− mice, we confirmed that Iba1-goat displayed elevated co-labeling with VP in the PVN (∼66% of cells) and SON (∼37% of cells), with only ∼10–12% of Iba1-goat+ cells co-labeling with CX3CR1-GFP expression in both the PVN and SON, and with the remainder of the Iba1+ cells in the PVN (∼21%) and SON (∼50%) labeling only with Iba1-goat (Fig. 4*C*). In contrast, using the same subjects, Iba1-chicken displayed a different pattern of colocalization with these markers: <5% of the cells labeled by Iba1-chicken colocalized with VP in both the PVN and SON, and ∼88 and 93% of the Iba1-chicken-labeled cells colocalized with CX3CR1-GFP, in the PVN and SON, respectively (Fig. 4*D*). Importantly, this effect is not due to differences in staining robustness of VP+ cells (Fig. 4-1). The remaining cells that were labeled by only Iba1-chicken made up ∼10 and 5% of the total cells labeled by Iba1-chicken in the PVN and SON, respectively. These remaining cells labeled with only Iba1 and not co-labeled with CX3CR1-GFP or VP may be labeling barrier-associated macrophages, and in the case of Iba1-goat, also OT cells.

10.1523/ENEURO.0323-25.2025.f4-1 Figure 4-1 **Staining robustness of VP cells is not different between Iba1-goat and Iba1-chicken samples used in experiments.** A) The number of VP + cells within the region of interest is not different between Iba1-goat and Iba1-chicken samples, although the SON had fewer overall VP + cells than the PVN in both the Iba1-goat and Iba1-chicken samples, due to a smaller ROI size *(significant main effect of Brain Region; F(1,2)* *=* *66.94, p* *=* *0.0146).* B) The density of VP + cells within the region of interest is not different between Iba1-goat and Iba1-chicken samples and nor is it different by brain region. C) The number of Iba1 + cells colocalized with VP + cells is significantly increased in Iba1-goat samples, whereas the colocalization of these cell types in the Iba1-chicken samples is close to zero *(significant main effect of Iba1 Species; F(1,2)* *=* *119.3, p* *=* *0.0083)*. D) Similarly, the density of Iba1 + cells colocalized with VP + cells is significantly increased in Iba1-goat samples, whereas the density of colocalization of these cell types in the Iba1-chicken samples is close to zero *(significant main effect of Iba1 Species; F(1,2)* *=* *612.6, p* *=* *0.0016)*. Data are mean ± SEM; *p < 0.05. Download Figure 4-1, TIF file.

### The irregular staining patterns of Iba1-goat are species-specific and are not observed in Wistar rats

**Figure 5.**
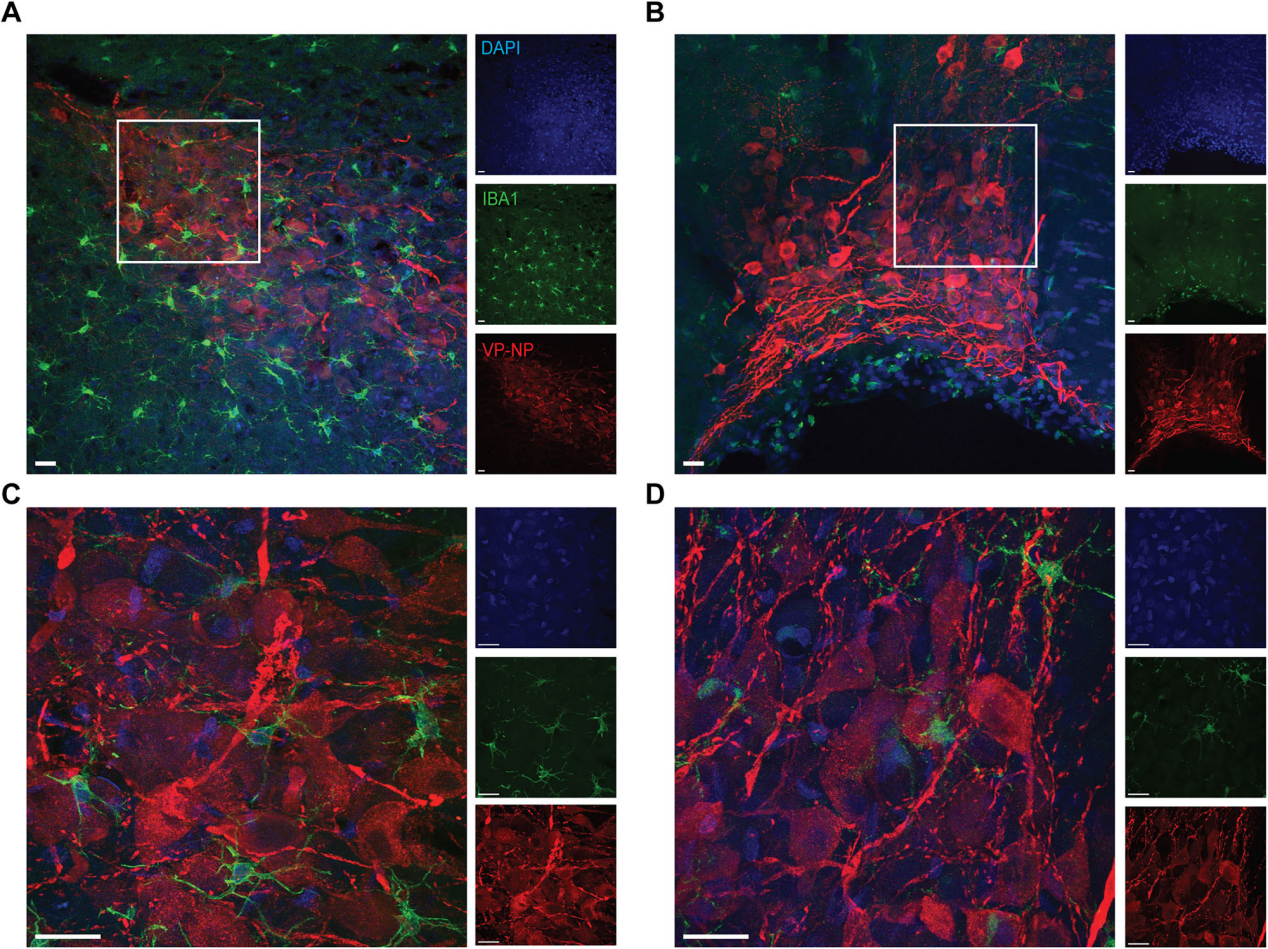
In Wistar rats, Iba1-goat displays normal labeling of microglial cells. ***A, D***, Representative images of the PVN (***A***) and SON (***B***) taken at 20×, as well as insets highlighting where higher-magnification images were taken at 63× of the PVN (***C***) and SON (***D***). Scale bar, 20 µm. Blue, DAPI; green, Iba1-goat; red, VP. The white boxes in ***A*** and ***B*** denote the location of 63× imaging.

In order to understand if this irregular staining pattern of Iba1-goat is specific to mice, we used samples from adult Wistar rats to perform IHC for anti-Iba1-goat and VP. Interestingly, in both the PVN and SON of rats, VP staining and anti-Iba1-goat staining were observed to be normal (Fig. 5). These qualitative results suggest that the anti-Iba1-goat antibody (Fujifilm WAKO Pure Chemical Corporation catalog #011-27991, RRID: AB_2935833) displays irregular staining in a species-specific manner.

## Discussion

The goal of this manuscript was to evaluate the efficacy of microglial cell labeling by three commonly used anti-Iba1 antibodies made in three separate host species when compared with other conventional microglial markers, i.e., anti-P2RY12 antibody and CX3CR1-GFP reporter. A secondary goal of this manuscript was to raise awareness of the importance of antibody validation in scientific research. The validation of antibodies is crucial prior to experimental procedures in order to avoid erroneous results and misinterpretation of scientific phenomena. Furthermore, it is important to validate antibody efficacy when using different lots of antibodies in order to ensure that results are consistent across batches. Although anti-Iba1 is one of the most commonly used antibodies for labeling microglial cells, we find in this study that the use of a specific version of this antibody made in goat, across multiple different lots (RRID: AB_2935833; Lots SKK1868, LEG4278, and WTQ1615), displays erroneous cell labeling of nonmicroglial cells in two brain regions, the PVN and the SON. These regions are also the primary source of vasopressin and oxytocin production, and further investigation into this phenomenon revealed that the erroneous cell labeling colocalized most highly with vasopressin neurons in these regions.

While multiple recent studies have used anti-Iba1-goat in C57BL/6 mice and did not report this erroneous cell labeling (Hampton et al., 2020; Iguchi et al., 2023; Cao et al., 2024; Lee et al., 2025; Soares et al., 2025; Wang et al., 2025), these papers mostly focused on hippocampal and cortical brain regions (which exhibited normal staining in our hands as well; Fig. 1-1), and did not investigate microglia within the PVN and SON regions of the hypothalamus using this antibody. Furthermore, it is possible that any lab that has previously attempted to utilize this anti-Iba1-goat antibody and found strange staining in the PVN and SON pivoted to use anti-Iba1 made in another host species or another microglial marker entirely, which is perhaps why this phenomenon has not been reported previously. While this antibody seems to have efficacy issues in mouse tissue in these regions, these issues do not arise in Wistar rats (Fig. 5), suggesting that the cross-reactivity of this antibody with vasopressin-expressing principal neurons in the PVN and SON is specific to mice. Interestingly, previous work in the field has shown that the amino acid sequence identity of Iba1 is 91.55% identical between mice and rats (Ohsawa et al., 2000). While the goal of this manuscript was not to investigate the reason why this anti-Iba1-goat antibody seems to have cross-reactivity with vasopressin-expressing cells in the PVN and SON of C57BL/6 mice, this mechanism would be interesting to investigate in future work, as well as whether this phenomenon occurs in other mouse strains. One potential reason that this labeling may occur with Iba1-goat is due to the possibility of cross-reactivity through the recognition of multiple epitopes. When comparing the amino acid sequences of Iba1 with vasopressin and oxytocin (which are ∼80% homologous with each other) within a mouse, we find that Iba1 is ∼30% homologous with vasopressin and oxytocin. It is possible that this weak, although significant, homology may explain why Iba1-goat could cross-react with vasopressin and oxytocin (Yu et al., 2004). Alternatively, or perhaps in addition, the cross-reactivity may be related to the differences in epitope reactivity when using polyclonal antibody production.

In order to understand if the observation of erroneous anti-Iba1-goat staining was a developmental phenomenon or was consistent across the lifespan, both pups and adults of both sexes were used in the first two experiments when comparing this anti-Iba1-goat antibody to alternative microglial markers (Fig. 1) or with other anti-Iba1 antibodies made in other host species (Fig. 2). In both cases, and in both the PVN and the SON, there were clear increases in the densities of cell labeling by anti-Iba1-goat compared with other microglial markers. These qualitative disparities were substantiated by our quantitative data, supporting the idea that this anti-Iba1-goat antibody labels more than just microglial cells in the PVN and SON across the lifespan in C57BL/6 mice. Interestingly, we observed some slight differences in cell labeling densities among the other anti-Iba1 species: in the SON (but not the PVN) of pups, Iba1-chicken displays a significant increase in the levels of colocalization with CX3CR1-GFP compared with the other anti-Iba1 species (Fig. 2*C*), and the same phenomenon was observed in the PVN of adult mice (but not in the SON). This suggests that, in some cases, anti-Iba1-chicken may provide the most similar cell labeling results to CX3CR1-GFP, a transgenic reporter for microglia.

Despite all species’ versions of the Iba1 antibody utilizing the same immunogens based on the publicly available information, it is possible that once the immunogen enters the species from which the Iba1 antibody is derived, the individual organisms produce various antibodies in response to that immunogen. Interestingly, both the Iba1-goat and Iba1-rabbit variants are polyclonal antibodies, whereas the Iba1-chicken is a monoclonal recombinant antibody. Thus, the Iba1-chicken antibody is likely more precise and reproducible between lots, as monoclonal antibodies are composed of a single IgG against one epitope. In contrast, polyclonal antibodies contain a heterologous mixture of IgGs against the whole immunogen, suggesting that the specific epitopes that the Iba1 antibody interacts with between the goat and rabbit variants may differ from each other, and from the chicken variant. Due to this heterogeneity, one would think that there might be lot-to-lot variability in these polyclonal antibodies, but in this study, we consistently found extraneous labeling of vasopressin neurons by Iba1-goat throughout all lots that were used. In summary, while the same immunogen may be utilized to produce anti-Iba1 antibodies in different species, polyclonal antibodies made in different species may produce different antibodies against various epitopes, resulting in differences in immunohistochemistry results.

Importantly, when considering the specificity of Iba1 compared with CX3CR1 in brain tissue, Iba1 labels monocytes/macrophages generally, whereas CX3CR1 is more highly expressed by microglia compared with perivascular macrophages (Mondo et al., 2020; Mills et al., 2025). When comparing the colocalization of Iba1-goat or Iba1-chicken with CX3CR1-GFP or VP, we found that there were a portion of cells in both the PVN and SON that have only Iba1 labeling (Fig. 4). In the context of Iba1-goat, this may be predominately due to partial colocalization with oxytocin (Fig. 3*C*), but this staining may also represent peripheral macrophages, especially as the regions analyzed in this study are bordering the ventricles. It has been previously shown that Iba1 not only primarily labels microglia but also labels other myeloid cells such as macrophages (Ito et al., 1998; Unger et al., 2018; Kenkhuis et al., 2022), which could explain the 5–10% of Iba1-chicken+ cells that did not colocalize with CX3CR1-GFP in our data (Fig. 4*D*).

Our results indicate that it is important to validate antibody efficacy when using new antibodies prior to beginning experimental data collection in order to ensure that cell labeling is both specific and accurate. This concept is particularly important when using new antibodies for the same molecular target (in this case, anti-Iba1) made in different host species. While we did not observe that specific lots of anti-Iba1-goat produced these erroneous effects, it is still possible that these errors may arise in other antibodies in specific lots.

Overall, not all commercially available antibodies for anti-Iba1 detect microglial cells with the specificity as advertised in C57BL/6 mice. Thus, researchers should use caution when employing new antibodies in experiments, validating that new antibodies function as expected in all regions of interest by using alternative markers for the cell type of interest that have already been validated by the lab. Furthermore, the results of this study emphasize the importance of validating all antibodies prior to experimentation, as this may not be the only case in which there is a species- and region-specific cross-reactivity of an antibody with another cell type.

## Synthesis

Reviewing Editor: Eran Perlson, Tel Aviv University Decisions are customarily a result of the Reviewing Editor and the peer reviewers coming together and discussing their recommendations until a consensus is reached. When revisions are invited, a fact-based synthesis statement explaining their decision and outlining what is needed to prepare a revision will be listed below. The following reviewer(s) agreed to reveal their identity: NONE.

This article addresses an important methodological issue in immunocytochemistry and neuroscience, the risk of misinterpretation in antibody-based labeling due to non-specific immunoreactivity. The study highlights concerns with commercially available Iba1 antibodies, commonly used to label microglia, which were also found to label nonapeptide cell bodies in the mouse hypothalamus. This observation underscores the potential for misleading results when relying on antibodies without sufficient validation, and the authors propose a useful algorithm for minimizing such errors. The manuscript is well organized, clearly written, and presents convincing evidence supporting its conclusions. While the report is brief and methodological in nature, it provides valuable insights that could benefit other researchers.

That said, several improvements would strengthen the manuscript and increase its informativeness:

1. Add a brief description of Iba1, including its biochemical/functional properties and why it is widely used as a microglia/macrophage marker, to provide context.

2. Include, if available, information from antibody manufacturers on the immunogens used to generate the different Iba1 antibodies.

3. Clarify the x-axis labeling in Figure 2 to indicate which antibody (chicken, goat, rabbit) corresponds to which data, and consider making the figure more color-blind friendly.

4. Provide representative z-stack or rotated confocal images to confirm true co-localization between Iba1 and OT/AVP labeling, distinguishing it from close proximity.

5. Report whether the total numbers of OT/AVP cells were comparable across different Iba1 stains, as differences in staining robustness (e.g., goat vs. chicken antibodies) could influence interpretation.

6. For Figure 5, include lower-magnification rat images comparable to the mouse data to clearly demonstrate the absence of co-localization.

7. Expand the discussion by addressing:

-The degree of amino acid sequence identity of Iba1 between mouse and rat.

-The possibility that different antibodies recognize distinct epitopes of Iba1.

-Whether similarities between Iba1 epitopes and those of other hypothalamic proteins could explain unexpected cross-reactivity.

## Author Response

Synthesis Statement for Author (Required):

This article addresses an important methodological issue in immunocytochemistry and neuroscience, the risk of misinterpretation in antibody-based labeling due to non-specific immunoreactivity. The study highlights concerns with commercially available Iba1 antibodies, commonly used to label microglia, which were also found to label nonapeptide cell bodies in the mouse hypothalamus. This observation underscores the potential for misleading results when relying on antibodies without sufficient validation, and the authors propose a useful algorithm for minimizing such errors. The manuscript is well organized, clearly written, and presents convincing evidence supporting its conclusions. While the report is brief and methodological in nature, it provides valuable insights that could benefit other researchers.

That said, several improvements would strengthen the manuscript and increase its informativeness:

1. Add a brief description of Iba1, including its biochemical/functional properties and why it is widely used as a microglia/macrophage marker, to provide context.

We thank the reviewers for this insightful idea as an addition to our manuscript. This context has now been provided within the manuscript in the introduction section. The provided context reads: "Iba1, or ionized calcium binding adaptor molecule 1, has been shown to be expressed specifically in monocyte/macrophage cell lineages, and functions as an adapter molecule that mediates calcium signals in these cells (Ito et al., 1998; Okere &Kaba, 2000). While other microglial markers, like CR3 or ED2, are only expressed in subsets of microglia, Iba1 is expressed by all microglial subpopulations, making it the most popular pan-microglial marker (Ito et al., 1998; Shapiro et al., 2009)." 2. Include, if available, information from antibody manufacturers on the immunogens used to generate the different Iba1 antibodies.

We investigated the publicly available immunogen information and found that all the different species of Iba1 targeted the immunogen "synthetic peptide C-terminal of Iba1". Upon reaching out to both WAKO and Synaptic Systems, we only received the specific immunogens for the Synaptic Systems Iba1-Chicken antibody, which is "C-terminal AA: PTGPPAKKAISELP from rat Iba1". Unfortunately, WAKO's response was that the "antigen used for the Iba1 goat antibody is a C-terminal homologous peptide of Iba1, but the specific sequence information is confidential. The same antigen sequence is used for both the goat and rabbit antibodies. After reviewing the article, it appears this phenomenon occurs only under very specific conditions and is not expected to be an issue for the majority of researchers." While we disagree with the sentiment that this would not be an issue for the majority of researchers, we feel it would not be appropriate to only include immunogen information from one manufacturer if the other manufacturer is not open to sharing this information. Thus, we cannot conjecture what specifically is the reason that only one subtype of this Iba1 antibody is leading to the extraneous labeling. We have, however, added to the manuscript an additional point about immunogens within the discussion, as related to comment 7b (see below).

3. Clarify the x-axis labeling in Figure 2 to indicate which antibody (chicken, goat, rabbit) corresponds to which data and consider making the figure more color-blind friendly.

We appreciate this sentiment from the reviewers and have chosen colors that are more color-blind friendly for this figure. Regarding the antibody species that correspond to the data on the x-axis, if we were to indicate the species for each Iba1 antibody in the x-axis, the labels would be quite long, so we have used colors that correspond to the figure legend to notate which Iba1 antibody corresponds to which data.

4. Provide representative z-stack or rotated confocal images to confirm true co-localization between Iba1 and OT/AVP labeling, distinguishing it from close proximity.

We have provided representative z-stack images to confirm true co-localization between Iba1 and OT/AVP labeling as a supplement, titled Figure 3-1.

5. Report whether the total numbers of OT/AVP cells were comparable across different Iba1 stains, as differences in staining robustness (e.g., goat vs. chicken antibodies) could influence interpretation.

We have now included supplemental graphs, titled Figure 4-1, which includes a comparison of the cell counts and cell densities of vasopressin cells within the regions of interest, as well as cell counts and cell densities of Iba1+ cells colocalized with vasopressin cells. The data indicates that the average number and density of vasopressin cells within each region of interest is comparable between Iba1-goat and Iba1-chicken trials, whereas the average number and density of Iba1+ cells colocalized with vasopressin cells is significantly different between the two Iba1 variants. We have also added this phrasing to the manuscript in the results section: "Importantly, this effect is not due to differences in staining robustness of VP+ cells (Fig. 4-1)." 6. For Figure 5, include lower-magnification rat images comparable to the mouse data to clearly demonstrate the absence of co-localization.

We have now added lower-magnification rat images to be more comparable to the mouse data and to clearly demonstrate the absence of co-localization in rats.

7. Expand the discussion by addressing: a. The degree of amino acid sequence identity of Iba1 between mouse and rat.

We thank the reviewers for this excellent suggestion. We have now added to the discussion (second paragraph) the following statement: "Interestingly, previous work in the field has shown that the amino acid sequence identity of Iba1 is 91.55% identical between mice and rats (Ohsawa et al., 2000)." We also verified these results by using Protein BLAST, and a screenshot of my search results is attached for reference. b. The possibility that different antibodies recognize distinct epitopes of Iba1.

We have now added the following to the discussion in the fourth paragraph: "Despite all species' versions of the Iba1 antibody utilizing the same immunogens based on the publicly available information, it is possible that once the immunogen enters the species from which the Iba1 antibody is derived, the individual organisms produce various antibodies in response to that immunogen. Interestingly, both the Iba1-goat and Iba1-rabbit variants are polyclonal antibodies, whereas the Iba1-chicken is a monoclonal recombinant antibody. Thus, the Iba1-chicken antibody is likely more precise and reproducible between lots, as monoclonal antibodies are composed of a single IgG against one epitope. In contrast, polyclonal antibodies contain a heterologous mixture of IgGs against the whole immunogen, suggesting that the specific epitopes that the Iba1-antibody interacts with between the goat and rabbit variants may differ from each other, and from the chicken variant. Due to this heterogeneity, one would think that there might be lot-to-lot variability in these polyclonal antibodies, but in this study, we consistently found extraneous labeling of vasopressin neurons by Iba1-goat throughout all lots that were used. In summary, while the same immunogen may be utilized to produce anti-Iba1 antibodies in different species, polyclonal antibodies made in different species may produce different antibodies against various epitopes, resulting in differences in immunohistochemistry results." c. Whether similarities between Iba1 epitopes and those of other hypothalamic proteins could explain unexpected cross-reactivity.

We thank the reviewers for prompting us to investigate this idea and have now discovered that Iba1 exhibits ~30% homology with vasopressin and oxytocin in mice (see screen captures below). We have now added the following information to our discussion in the second paragraph: "One potential reason that this labeling may occur with Iba1-goat is due to the possibility of cross-reactivity through the recognition of multiple epitopes. When comparing the amino acid sequences of Iba1 with vasopressin and oxytocin (which are ~80% homologous with each other) within mouse, we find that Iba1 is ~30% homologous with vasopressin and oxytocin. It is possible that this weak, although significant, homology may explain why Iba1-goat could cross-react with vasopressin and oxytocin (Yu et al., 2004). Alternatively, or perhaps in addition, the cross-reactivity may be related to the differences in epitope reactivity when using polyclonal antibody production." Iba1 mouse significant alignments for mouse NP-VP and mouse NP-OT sequence alignment

## References

[B1] Ben-Barak Y, Russell JT, Whitnall MH, Ozato K, Gainer H (1985) Neurophysin in the hypothalamo-neurohypophysial system. I. Production and characterization of monoclonal antibodies. J Neurosci 5:81–97. 10.1523/JNEUROSCI.05-01-00081.1985 3880813 PMC6565074

[B2] Bolton JL, et al. (2022) Early stress-induced impaired microglial pruning of excitatory synapses on immature CRH-expressing neurons provokes aberrant adult stress responses. Cell Rep 38:110600. 10.1016/j.celrep.2022.110600 35354026 PMC9014810

[B3] Cao X, et al. (2024) HCN channels in the lateral habenula regulate pain and comorbid depressive-like behaviors in mice. CNS Neurosci Ther 30:e14831. 10.1111/cns.14831 38961317 PMC11222070

[B4] Cronk JC, et al. (2018) Peripherally derived macrophages can engraft the brain independent of irradiation and maintain an identity distinct from microglia. J Exp Med 215:1627–1647. 10.1084/jem.20180247 29643186 PMC5987928

[B5] Gómez Morillas A, Besson VC, Lerouet D (2021) Microglia and neuroinflammation: what place for P2RY12? Int J Mol Sci 22:1636. 10.3390/ijms22041636 33561958 PMC7915979

[B6] Hammond TR, et al. (2019) Single-cell RNA sequencing of microglia throughout the mouse lifespan and in the injured brain reveals complex cell-state changes. Immunity 50:253–271.e6. 10.1016/j.immuni.2018.11.004 30471926 PMC6655561

[B7] Hampton DW, Amor S, Story D, Torvell M, Bsibsi M, van Noort JM, Chandran S (2020) HspB5 activates a neuroprotective glial cell response in experimental tauopathy. Front Neurosci 14:574. 10.3389/fnins.2020.00574 32595446 PMC7300208

[B8] Iguchi A, et al. (2023) INPP5D modulates TREM2 loss-of-function phenotypes in a β-amyloidosis mouse model. iScience 26:106375. 10.1016/j.isci.2023.106375 37035000 PMC10074152

[B9] Ito D, Imai Y, Ohsawa K, Nakajima K, Fukuuchi Y, Kohsaka S (1998) Microglia-specific localisation of a novel calcium binding protein, Iba1. Mol Brain Res 57:1–9. 10.1016/S0169-328X(98)00040-0 9630473

[B10] Jurga AM, Paleczna M, Kuter KZ (2020) Overview of general and discriminating markers of differential microglia phenotypes. Front Cell Neurosci 14:198. 10.3389/fncel.2020.00198 32848611 PMC7424058

[B11] Kenkhuis B, Somarakis A, Kleindouwel LRT, van Roon-Mom WMC, Höllt T, van der Weerd L (2022) Co-expression patterns of microglia markers Iba1, TMEM119 and P2RY12 in Alzheimer’s disease. Neurobiol Dis 167:105684. 10.1016/j.nbd.2022.105684 35247551

[B12] Lee SH, Bae E-J, Park SJ, Lee S-J (2025) Microglia-driven inflammation induces progressive tauopathies and synucleinopathies. Exp Mol Med 57:1017–1031. 10.1038/s12276-025-01450-z 40307569 PMC12130470

[B13] Lenz KM, Nugent BM, Haliyur R, McCarthy MM (2013) Microglia are essential to masculinization of brain and behavior. J Neurosci 33:2761–2772. 10.1523/JNEUROSCI.1268-12.2013 23407936 PMC3727162

[B14] Meliopoulos VA, Schultz-Cherry S (2018) Although it’s painful: the importance of stringent antibody validation. PLoS Pathog 14:e1006701. 10.1371/journal.ppat.1006701 29300781 PMC5754129

[B15] Mills WA, et al. (2025) Microglial cyclooxygenase-1 modulates cerebral capillary basal tone in vivo in mice. Nat Commun 16:5704. 10.1038/s41467-025-60753-x 40595608 PMC12216979

[B16] Miyamoto A, Wake H, Ishikawa AW, Eto K, Shibata K, Murakoshi H, Koizumi S, Moorhouse AJ, Yoshimura Y, Nabekura J (2016) Microglia contact induces synapse formation in developing somatosensory cortex. Nat Commun 7:1. 10.1038/ncomms12540 PMC5007295 27558646

[B17] Mondo E, Becker SC, Kautzman AG, Schifferer M, Baer CE, Chen J, Huang EJ, Simons M, Schafer DP (2020) A developmental analysis of juxtavascular microglia dynamics and interactions with the vasculature. J Neurosci 40:6503–6521. 10.1523/JNEUROSCI.3006-19.2020 32661024 PMC7486666

[B18] Nelson LH, Peketi P, Lenz KM (2021) Microglia regulate cell genesis in a sex-dependent manner in the neonatal hippocampus. Neuroscience 453:237–255. 10.1016/j.neuroscience.2020.10.009 33129890

[B19] Ohsawa K, Imai Y, Kanazawa H, Sasaki Y, Kohsaka S (2000) Involvement of Iba1 in membrane ruffling and phagocytosis of macrophages/microglia. J Cell Sci 113:3073–3084. 10.1242/jcs.113.17.3073 10934045

[B20] Ohsawa K, Imai Y, Sasaki Y, Kohsaka S (2004) Microglia/macrophage-specific protein Iba1 binds to fimbrin and enhances its actin-bundling activity. J Neurochem 88:844–856. 10.1046/j.1471-4159.2003.02213.x 14756805

[B21] Okere CO, Kaba H (2000) Heterogenous immunohistochemical expression of microglia-specific ionized calcium binding adaptor protein (Iba1) in the mouse olfactory bulb. Brain Res 877:85–90. 10.1016/S0006-8993(00)02656-1 10980247

[B22] Paolicelli RC, et al. (2011) Synaptic pruning by microglia is necessary for normal brain development. Science 333:1456–1458. 10.1126/science.1202529 21778362

[B23] Schafer DP, Lehrman EK, Kautzman AG, Koyama R, Mardinly AR, Yamasaki R, Ransohoff RM, Greenberg ME, Barres BA, Stevens B (2012) Microglia sculpt postnatal neural circuits in an activity and complement-dependent manner. Neuron 74:691–705. 10.1016/j.neuron.2012.03.026 22632727 PMC3528177

[B24] Shapiro LA, Perez ZD, Foresti ML, Arisi GM, Ribak CE (2009) Morphological and ultrastructural features of Iba1-immunolabeled microglial cells in the hippocampal dentate gyrus. Brain Res 1266:29–36. 10.1016/j.brainres.2009.02.031 19249294 PMC2677570

[B25] Soares AR, Garcia-Rivas V, Fai C, Thomas M, Zheng X, Picciotto MR, Mineur YS (2025) Sex differences in the microglial response to stress and chronic alcohol exposure in mice. Biol Sex Differ 16:19. 10.1186/s13293-025-00701-y 40038827 PMC11881309

[B26] Unger MS, Schernthaner P, Marschallinger J, Mrowetz H, Aigner L (2018) Microglia prevent peripheral immune cell invasion and promote an anti-inflammatory environment in the brain of APP-PS1 transgenic mice. J Neuroinflammation 15:274. 10.1186/s12974-018-1304-4 30241479 PMC6151006

[B27] Verbalis JG, Robinson AG (1983) Characterization of neurophysin-vasopressin prohormones in human posterior pituitary tissue. J Clin Endocrinol Metab 57:115–123. 10.1210/jcem-57-1-115 6406529

[B28] Wang Z, et al. (2025) Early life high fructose exposure disrupts microglia phagocytosis and impedes neurodevelopment. Nature 644:759–768. 10.1038/s41586-025-09098-5 40500435 PMC7617807

[B29] Yu H, Luscombe NM, Lu HX, Zhu X, Xia Y, Han J-DJ, Bertin N, Chung S, Vidal M, Gerstein M (2004) Annotation transfer between genomes: protein–protein interologs and protein–DNA regulogs. Genome Res 14:1107–1118. 10.1101/gr.1774904 15173116 PMC419789

[B30] Zhao X-F, Alam MM, Liao Y, Huang T, Mathur R, Zhu X, Huang Y (2019) Targeting microglia using Cx3cr1-Cre lines: revisiting the specificity. eNeuro 6:ENEURO.0114-19.2019. 10.1523/ENEURO.0114-19.2019 PMC6620394 31201215

