# Supplementary figures and images for: A Common Iba1 Antibody Labels Vasopressin Neurons in Mice
Source: eNeuro. 2026 Jan 30;13(2):ENEURO.0323-25.2025. doi: 10.1523/ENEURO.0323-25.2025 (PMC12871090; doi:10.1523/ENEURO.0323-25.2025)

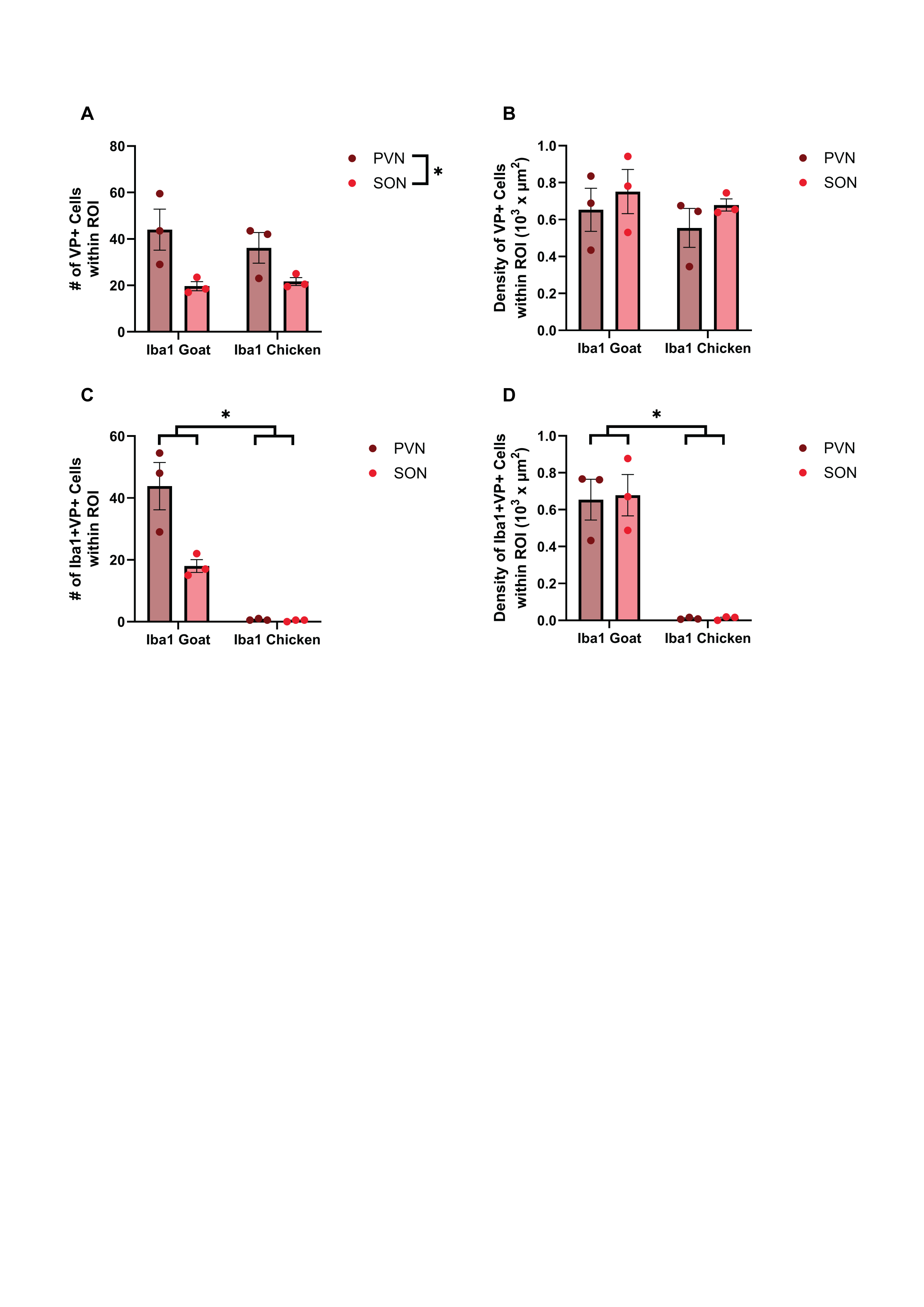

Supplement: Figure 1-1 — Excessive Iba1-goat staining is confined to specific regions of the mouse brain. A-B) Representative images of the parietal cortex in A) pups and B) adults. C-D) Representative images of the CA1 of the hippocampus in (C) pups and (D) adults. Scale bars = 20 μm, blue = DAPI, green = CX3CR1, red = Iba1-goat, magenta = P2RY12. Download Figure 1-1, TIF file. [file eneuro-13-ENEURO.0323-25.2025-s001.tif]

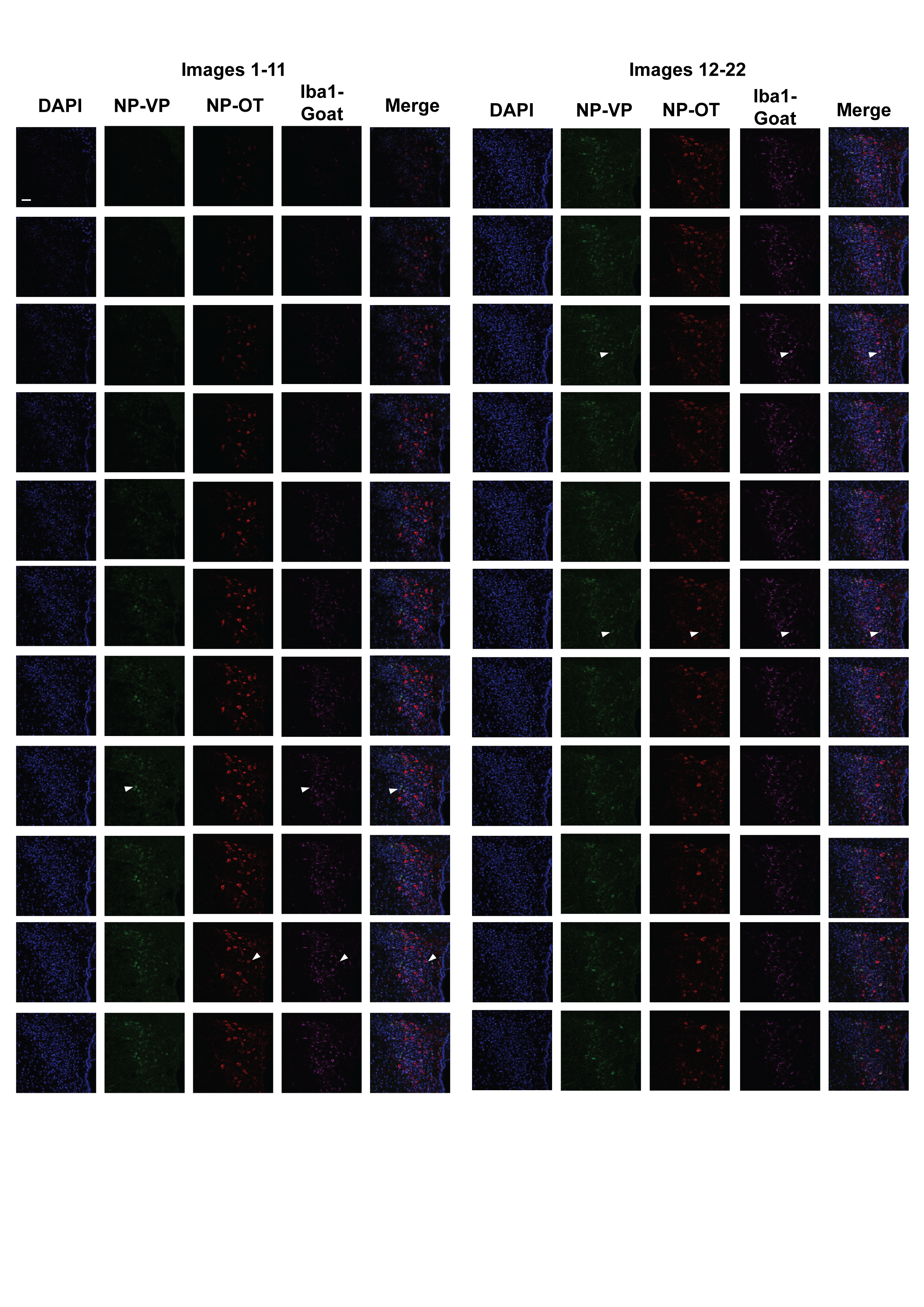

Supplement: Figure 3-1 — Representative z-stack image sequence to confirm true colocalization between Iba1 and OT and VP. Corresponds to the PVN samples from Figure 3. Scale bar = 20 μm, blue = DAPI, green = VP, red = OT, magenta = Iba1-goat. White arrowheads represent examples of colocalization between Iba1-goat, and VP and/or OT. Download Figure 3-1, TIF file. [file eneuro-13-ENEURO.0323-25.2025-s002.tif]

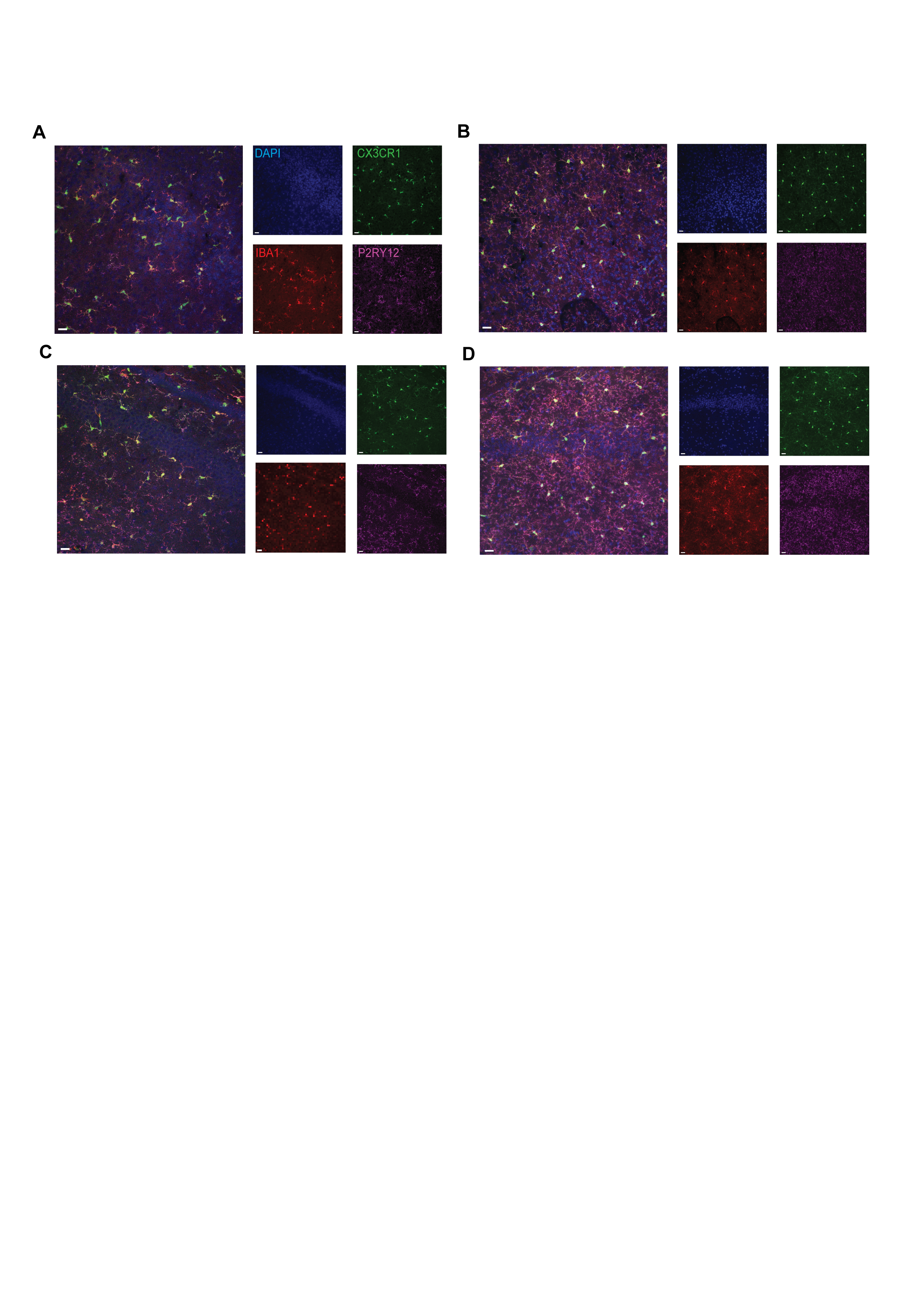

Supplement: Figure 4-1 — Staining robustness of VP cells is not different between Iba1-goat and Iba1-chicken samples used in experiments. A) The number of VP + cells within the region of interest is not different between Iba1-goat and Iba1-chicken samples, although the SON had fewer overall VP + cells than the PVN in both the Iba1-goat and Iba1-chicken samples, due to a smaller ROI size (significant main effect of Brain Region; F(1,2) = 66.94, p = 0.0146). B) The density of VP + cells within the region of interest is not different between Iba1-goat and Iba1-chicken samples and nor is it different by brain region. C) The number of Iba1 + cells colocalized with VP + cells is significantly increased in Iba1-goat samples, whereas the colocalization of these cell types in the Iba1-chicken samples is close to zero (significant main effect of Iba1 Species; F(1,2) = 119.3, p = 0.0083). D) Similarly, the density of Iba1 + cells colocalized with VP + cells is significantly increased in Iba1-goat samples, whereas the density of colocalization of these cell types in the Iba1-chicken samples is close to zero (significant main effect of Iba1 Species; F(1,2) = 612.6, p = 0.0016). Data are mean ± SEM; *p < 0.05. Download Figure 4-1, TIF file. [file eneuro-13-ENEURO.0323-25.2025-s003.tif]
